# Schiff bases of indoline-2,3-dione (isatin) with potential antiproliferative activity

**DOI:** 10.1186/1752-153X-6-49

**Published:** 2012-05-30

**Authors:** Tarek Aboul-Fadl, Awwad A Radwan, Mohamed I Attia, Abdullah Al-Dhfyan, Hatem A Abdel-Aziz

**Affiliations:** 1 Department of Pharmaceutical Chemistry, College of Pharmacy, King Saud University, P.O. Box 2457, Riyadh, 11451, Saudi Arabia; 2 Faculty of Pharmacy, Assiut University, Assiut, 71526, Egypt; 3 Kayyali Chair, Pharmaceutical Technology center, College of Pharmacy, King Saud University, P.O. Box 2457, Riyadh, 11451, Saudi Arabia; 4 Stem Cell Therapy Program, King Faisal Specialized Hospital and Research Center, P.O. Box 3354, Riyadh, 11211, Saudi Arabia

**Keywords:** Isatin, Schiff bases, Combinatorial library, Antiproliferative, Pharmacophore

## Abstract

****Background**:**

Cancer is one of the most dreaded diseases and it is a leading cause of mankind death worldwide. Recent reports documented a remarkable antiproliferative activity of isatin nucleus against various cancer cell lines. The current work describes the antiproliferative activity of Schiff bases of combinatorial mixtures of the isatin derivatives **M1-M22** as well as the individual compounds **1-11(A-K)** of these combinatorial mixtures.

****Results**:**

The designed combinatorial library composed from eleven hydrazides **A-K** and eleven isatin derivatives **1-11** has been synthesized to formally generate 22 mixtures, **M1-M22** of 121 Schiff bases, and their antiproliferative activity against K562 chronic myelogenous leukemia cells was evaluated. The indexed method of analysis of the prepared library was applied to elucidate the active components in the tested mixtures **M1-M22**. The predictions from the crossing procedure was validated through evaluation of the antiproliferative activity of individual compounds **1-11(A-K)** of the library. Individual compounds **1-11(A-K)** were also evaluated against the non-tumorigenic MCF-12A cell line to investigate their selectivity. A pharmacophore model was developed to further optimize the antiproliferative activity among this series of compounds.

****Conclusions**:**

Variable antiproliferative activity was revealed with the investigated mixtures **M1-M22** and the individual compounds **1-11(A-K)**. Most of the tested mixtures and several individual Schiff bases displayed high potency with IC_50_ values in the low micromolar range. A considerable selectivity of some individual compounds to the tumorigenic K562 cell line compared with the non-tumorigenic MCF-12A cell line was observed as indicated by their selectivity index (SI).

## Background

Cancer is one of the most dreaded diseases of mankind. It is a leading cause of death throughout the world, and currently, one in 4 deaths in the United States is due to cancer [1]. More than ten million new cancer cases occur annually, roughly half of which is in the developed countries, and the disease causes over six million deaths a year [2,3]. Unlimited and uncontrolled cell proliferation is obviously characteristics of tumor cells [4].

Despite several decades of intensive research, the long-term outlook for patients with aggressive cancer remains discouraging, and there is a need for innovative approaches to design anticancer drugs with reduced toxicity and improved therapeutic indices [5,6]. In recent years, compounds containing hydrazide or hydrazone moieties are attractive target compounds for new drug development because of their potentially versatile biological activities involving antiproliferative activities [7,8]. Several studies have been devoted to the antiproliferative activity of aroylhydrazone derivatives [7,9-12]. It was suggested that the antiproliferative activity of these hydrazones may be attributed to inhibition of kinases [13-15], or through generation of radicals and dissipation of the mitochondrial membrane potential [16].

Furthermore, there are significant reasons for investigating the antiproliferative activity of Schiff base derivatives of indolin-2,3-dione (isatin) as recent reports documented a remarkable antiproliferative activity of isatin nucleus against various cancer cell lines [17-22]. Isatins have multiple molecular mechanisms to exert their anticancer activity. Among which tyrosine kinase Inhibiton (TKIs), inhibition of cyclin-dependent kinases (CDKs) by binding to the ATP pocket and/or caspase inhibition [23-25].

In continuation to our synthetic work on Schiff bases of isatin with potential biological activity [26-32], the current work describes the antiproliferative activity of Schiff bases of combinatorial mixtures of the isatin derivatives **M1-M22** as well as the individual compounds **1-11(A-K)** of these combinatorial mixtures. Moreover, *Ligand based pharmacophore modeling* of these Schiff bases was conducted to evaluate the common features essential for activity and the hypothetical geometries adopted by these ligands in their most active conformers were undertaken.

## Results and discussion

The designed combinatorial library mixtures **M1-M22** of the Schiff bases as well as its individual compounds **A-K(1-11)** were already published [27,29,30]. They were obtained either by conventional or microwave assisted methods and are summarized in Table 1.

**Table 1:** Antiproliferative evaluation results of the synthesized combinatorial mixtures M1-M22 and schematic representation of an orthogonal deconvolution for prediction of the active compounds

**Hydrazides**		**A**	**B**	**C**	**D**	**E**	**F**	**G**	**H**	**I**	**J**	**K**		
**Isatins**		(2)	(3)	(4)	(5)	(6)	(7)	(8)	(9)	(10)	(11)	(12)	**Set 1**	**IC**_**50**_^**a**^** (μM)**
**1**	(13)	**A1**	**B1**	**C1**	**D1**	**E1**	**F1**	**G1**	**H1**	**I1**	**J1**	**K1**	**M1**	7.1 ± 0.04
**2**	(14)	**A2**	**B2**	**C2**	**D2**	**E2**	**F2**	**G2**	**H2**	**I2**	**J2**	**K2**	**M2**	9.76 ± 0.027
**3**	(15)	**A3**	**B3**	**C3**	**D3**	**E3**	**F3**	**G3**	**H3**	**I3**	**J3**	**K3**	**M3**	7.16 ± 0.069
**4**	(16)	**A4**	**B4**	**C4**	**D4**	**E4**	**F4**	**G4**	**H4**	**I4**	**J4**	**K4**	**M4**	6.63 ± 0.02
**5**	(17)	**A5**	**B5**	**C5**	**D5**	**E5**	**F5**	**G5**	**H5**	**I5**	**J5**	**K5**	**M5**	4.53 ± 0.028
**6**	(18)	**A6**	**B6**	**C6**	**D6**	**E6**	**F6**	**G6**	**H6**	**I6**	**J6**	**K6**	**M6**	4.48 ± 0.015
**7**	(19)	**A7**	**B7**	**C7**	**D7**	**E7**	**F7**	**G7**	**H7**	**I7**	**J7**	**K7**	**M7**	5.09 ± 0.01
**8**	(20)	**A8**	**B8**	**C8**	**D8**	**E8**	**F8**	**G8**	**H8**	**I8**	**J8**	**K8**	**M8**	^b^
**9**	(21)	**A9**	**B9**	**C9**	**D9**	**E9**	**F9**	**G9**	**H9**	**I9**	**J9**	**K9**	**M9**	9.29 ± 0.019
**10**	(22)	**A10**	**B10**	**C10**	**D10**	**E10**	**F10**	**G10**	**H10**	**I10**	**J10**	**K10**	**M10**	9.76 ± 0.018
**11**	(23)	**A11**	**B11**	**C11**	**D11**	**E11**	**F11**	**G11**	**H11**	**I11**	**J11**	**K11**	**M11**	14.57 ± 0.05
Set 2		**M12**	**M13**	**M14**	**M15**	**M16**	**M17**	**M18**	**M19**	**M20**	**M21**	**M22**		
IC_50_^a^ (μM)		12.02±0.026	^b^	22.6±0.038	4.85±0.018	10.1±0.05	^b^	9.64±0.003	21.06±0.02	^b^	^b^	4.51±0.003		

^a^IC_50_: concentration of the compound (μM) producing 50 % cell growth inhibition after 48 h of compound exposure, as determined by the WST-1 assay. Each experiment was run at least two times, and the results are presented as average values±standard deviation. ^b^Compounds or mixtures having IC_50_ value > 100 μM.

*In vitro* antiproliferative activity of the combinatorial mixtures **M1-M22** was carried out by use of WST-1 reagent for determination of IC_50_ for each mixture against K562 chronic myelogenous leukemia cells according to the protocol mentioned in the experimental section and results are given in Table 1. Variable antiproliferative activity was observed with the investigated mixtures in the following decreasing order: **M6 > M22 > M5 > M15 > M7 > M4 > M1 > M3 > M9 > M18 > M2 = M10 > M16 > M11 > M19 > M14** with IC_50_ range values from 4.48 to 22.6 μM. Other mixtures did not show significant antiproliferative activity against MCF-12A cell line (IC_50_ > 100 μM). The most active mixtures in the first set are mixtures **M5-M7** whereas in the second set are mixtures **M15, M18**, and **M22**. The indexed method of analysis of the prepared library was applied to elucidate the active components in the tested mixtures **M1-M22**. Intersection of the active rows **M1-M7**, and **M9-M11** with the active columns **M12, M14-M16, M18, M19**, and **M22** gave the location of the possible active components in these mixtures, distinguished by bold cell borders (see Additional file 1).

In order to confirm the reliability of the predictions from the crossing procedure, the synthesized individual compounds **A-K(1-11)** were also investigated against K562 cell line. From the scattered data given in Table 2, it was difficult to determine precisely the essential moieties in compounds **1-11(A-K)** required to elicit antiproliferative activity. A general conclusion, however, can be made about the SAR in the synthesized series of hydrazones **1-11(A-K)** that the integrated molecular structure features are responsible for the elucidated antiproliferative activity irrespective of the building blocks incorporated in individual molecules. According to the displayed antiproliferative activity of the title compounds

**Table 2:** Cytotoxicity activity of the synthesized individual compounds 1-11(A-K)

	**Compound/IC**_**50**_** (μM)**										
**Compound**	**A1**	**B1**	**C1**	**D1**	**E1**	**F1**	**G1**	**H1**	**I1**	**J1**	**K1**
**K562 cell line**	20.04 ± 0.07	42.3 ± 0.03	13.47 ± 0.038	11.5 ± 0.01	6 ± 0.007	7.7 ± 0.02	5.66 ± 0.11	15.37 ± 0.02	11.59 ± 0.034	> 100	> 100
**MCF-12A cell line**	69.23 ± 0.123	49.39 ± 0.16	> 300	> 300	> 300	64.31 ± 0.026	16.61 ± 0.181	37.12 ± 0.27	> 300	> 300	> 300
**SI**	3.45	1.17	> 22.27	> 26.1	> 50.00	8.4	2.9	2.42	> 25.88	**NA**	**NA**
**Compound**	**A2**	**B2**	**C2**	**D2**	**E2**	**F2**	**G2**	**H2**	**I2**	**J2**	**K2**
**K562 cell line**	49.13 ± 0.095	20.19 ± 0.09	29.82 ± 0.139	8.3 ± 0.003	7.76 ± 0.07	12.9 ± 0.002	5.72 ± 0.1	8.05 ± 0.035	8.67 ± 0.027	23.32 ± 0.32	> 100
**MCF-12A cell line**	32.55 ± 0.073	> 300	> 300	14.78 ± 0.4	> 300	31.39 ± 0.096	16.62 ± 0.182	11.67 ± 0.003	> 300	17.91 ± 0.62	> 300
**SI**	0.66	> 14.86	> 10.06	1.8	> 38.66	2.4	2.9	1.45	> 34.60	0.77	**NA**
**Compound**	**A3**	**B3**	**C3**	**D3**	**E3**	**F3**	**G3**	**H3**	**I3**	**J3**	**K3**
**K562 cell line**	32.37 ± 0.074	19.35 ± 0.019	14.86 ± 0.055	13.86 ± 0.01	7.13 ± 0.009	14 ± 0.08	9.88 ± 0.41	> 100	15.81 ± 0.11	19.75 ± 0.08	> 100
**MCF-12A cell line**	27.06 ± 0.272	> 300	> 300	258.09 ± 0.03	> 300	8.71 ± 0.17	13.22 ± 0.034	97.36 ± 0.062	27.87 ± 0.07	> 300	83.8± 0.039
**SI**	0.84	> 15.50	> 20.19	18.6	> 42.08	0.62	1.3	NA	1.76	> 15.19	**NA**
**Compound**	**A4**	**B4**	**C4**	**D4**	**E4**	**F4**	**G4**	**H4**	**I4**	**J4**	**K4**
**K562 cell line**	38.88 ± 0.138	51.51 ± 0.09	30.96 ± 0.2	9.98 ± 0.009	7.5 ± 0.03	16.28 ± 0.015	8.43 ± 0.163	24.21 ± 0.023	11.57 ± 0.051	14.52 ± 0.08	> 100
**MCF-12A cell line**	9.39 ± 0.152	> 300	> 300	> 300	> 300	50.85 ± 0.048	12.50 ± 0.216	> 300	> 300	12.74 ± 0.32	78.29 ± 0.31
**SI**	0.24	> 5.82	> 9.69	> 30	> 40.00	3.1	1.5	> 12.39	> 25.93	0.88	**NA**
**Compound**	**A5**	**B5**	**C5**	**D5**	**E5**	**F5**	**G5**	**H5**	**I5**	**J5**	**K5**
**K562 cell line**	> 100	7.3 ± 0.01	23.9 ± 0.26	8.67 ± 0.007	10.3 ± 0.001	10.4 ± 0.044	6.5 ± 0.001	5.88 ± 0.004	24.11 ± 0.255	6.99 ± 0.005	> 100
**MCF-12A cell line**	39.74 ± 0.073	> 300	> 300	80.05 ± 0.40	> 300	39.09 ± 0.24	9.1 ± 0.003	7.35 ± 0.094	> 300	5.6 ± 0.003	> 300
**SI**	**NA**	> 41.10	> 12.55	9.2	> 29.13	3.8	1.4	1.25	> 12.44	0.80	**NA**
**Compound**	**A6**	**B6**	**C6**	**D6**	**E6**	**F6**	**G6**	**H6**	**I6**	**J6**	**K6**
**K562 cell line**	> 100	> 100	> 100	8.87 ± 0.017	9.58 ± 0.03	13.5 ± 0.03	6.62 ± 0.22	6.49 ± 0.017	8.55 ± 0.06	7.19 ± 0.051	> 100
**MCF-12A cell line**	13.91 ± 0.003	> 300	> 300	134.18 ± 0.142	> 300	9.6 ± 0.15	13.05 ± 0.20	34.77 ± 0.062	26.15 ± 0.14	10.16 ± 1.2	> 300
**SI**	**NA**	**NA**	**NA**	15.1	> 31.32	0.71	2	5.36	3.06	1.41	**NA**
**Compound**	**A7**	**B7**	**C7**	**D7**	**E7**	**F7**	**G7**	**H7**	**I7**	**J7**	**K7**
**K562 cell line**	> 100	47.25 ± 0.027	> 100	9.1 ± 0.026	8.9 ± 0.02	12.86 ± 0.01	7.68 ± 0.34	23.67 ± 0.39	> 100	18.02 ± 0.13	> 100
**MCF-12A cell line**	11.03 ± 0.151	> 300	> 300	> 300	> 300	11.39 ± 0.25	12.98 ± 0.439	73.18 ± 0.072	> 300	> 300	61.34 ± 0.01
**SI**	NA	> 6.35	NA	> 33	> 33.71	0.89	1.7	3.09	**NA**	> 16.65	**NA**
**Compound**	**A8**	**B8**	**C8**	**D8**	**E8**	**F8**	**G8**	**H8**	**I8**	**J8**	**K8**
**K562 cell line**	> 100	27.23 ± 0.092	> 100	6.9 ± 0.001	10.34 ± 0.015	6.99 ± 0.007	15.12 ± 0.48	40.65 ± 0.24	> 100	45.51 ± 0.46	> 100
**MCF-12A cell line**	10.54 ± 0.157	> 300	> 300	> 300	> 300	21.29 ± 0.104	> 300	> 300	> 300	> 300	> 300
**SI**	**NA**	> 11.02	NA	> 43.5	> 29.01	3	> 19.8	> 7.38	**NA**	> 6.59	**NA**
**Compound**	**A9**	**B9**	**C9**	**D9**	**E9**	**F9**	**G9**	**H9**	**I9**	**J9**	**K9**
**K562 cell line**	90.67 ± 0.19	17.42 ± 0.05	> 100	6.2 ± 0.01	9.77 ± 0.01	6.77 ± 0.01	> 100	26.72 ± 0.38	31.19 ± 0.004	52.05 ± 0.15	> 100
**MCF-12A cell line**	14.48 ± 0.647	> 300	> 300	> 300	> 300	13.14 ± 0.114	> 300	> 300	> 300	> 300	5.88 ± 0.11
**SI**	0.16	> 17.22	**NA**	> 48.4	> 30.71	1.9	**NA**	> 11.23	> 9.62	> 5.76	**NA**
**Compound**	**A10**	**B10**	**C10**	**D10**	**E10**	**F10**	**G10**	**H10**	**I10**	**J10**	**K10**
**K562 cell line**	> 100	17.88 ± 0.062	> 100	8.9 ± 0.43	8.66 ± 0.06	6.3 ± 0.005	8.43 ± 0.18	23.34 ± 0.19	19.14 ± 0.07	> 100	88.85 ± 0.26
**MCF-12A cell line**	8.33 ± 0.044	> 300	> 300	12.57 ± 0.089	> 300	11.01 ± 0.11	14.19 ± 0.175	32.86 ± 0.065	> 300	> 300	51.02 ± 0.04
**SI**	**NA**	> 16.78	**NA**	1.4	> 34.64	1.7	1.7	1.41	> 15.67	**NA**	0.57
**Compound**	**A11**	**B11**	**C11**	**D11**	**E11**	**F11**	**G11**	**H11**	**I11**	**J11**	**K11**
**K562 cell line**	> 100	39.24 ± 0.006	> 100	9.11 ± 0.23	13.65 ± 0.52	5.92 ± 0.002	> 100	8.46 ± 0.27	> 100	> 100	> 100
**MCF-12A cell line**	7.88 ± 0.01	> 300	> 300	10.15 ± 0.072	> 300	12.46 ± 0.053	97.93 ± 0.16	29.84 ± 0.17	> 300	> 300	28.31 ± 0.14
**SI**	**NA**	> 7.65	**NA**	1.1	> 21.98	2.1	**NA**	3.53	**NA**	**NA**	**NA**

^a^IC_50_: concentration of the compound (μM) producing 50 % cell growth inhibition after 48 h of compound exposure, as determined by the WST-1 assay. Each experiment was run at least two times, and the results are presented as average values±standard deviation.

**1-11(A-K)** they can be divided into: highly active candidates with IC_50_ < 10 μM (**B5, D2-D11, E1-E4, E6, E7, E9, E10, F1, F8-F11, G1-G7, G10, H2, H5, H6, H11, I2, I6, J5, and J6**), moderately active candidates with IC_50_ < 20 μM (**B3, B9, B10, C1, C3, D1, D3, E5, E8, E11, F2-F7, G8, H1, I1, I3, I4, I10, J4, and J7**), weekly active with IC_50_ ~ 20 < 100 μM (**A1-A4, A9, B1, B2, B4, B7, B8, B11, C2, C4, C5, H4, H7-H10, I5, I9, J2, J8, J9, and K10**) and inactive with IC_50_ > 100 μM (rest of the compounds). It is clear that series **A** with isonicotinic acid hydrazide, **B** with nicotinic acid hydrazide, **C** with furan-2-carboxylic acid hydrazide and **K** with nalidixic acid hydrazide have the least contribution in antiproliferative activity.

Surprisingly, antiproliferative activity prediction of the individual compounds from the intersection of **M1-M11** and **M12-M22** was not consistent with the results achieved from practical investigations of individual compounds particularly in case of **A** and **K** series. This may be attributed to the additive contribution of the active components in their mixture that are effective than that tested separately.

To evaluate the selectivity of these individual compounds on the tumorigenic cells, their cytotoxicity was measured by cell growth inhibition assay against MCF-12A cell line. The MCF-12A cell line is a non-tumorigenic epithelial cell line established from tissue taken at reduction mammoplasty from a nulliparous patient with fibrocystic breast disease that contained focal areas of intraductal hyperplasia. The general *in vitro* cytotoxic evaluation of these synthesized compounds was carried out also by use of WST-1 reagent for determination of IC_50_ for each compound according to the protocol mentioned in the experimental section and results are given in Table 2. The selectivity index (SI) which represents IC_50_ for normal cell line/IC_50_ for cancerous cell line. As the SI demonstrates the differential activity of a compound, the greater the SI value is, the more selective it is. Variable selectivity was observed with the different investigated compounds (Table 2). Though it is difficult to contribute the selectivity pattern to either of the building blocks, the E series with benzofuran moiety revealed good selectivity pattern. Among the highly active candidates, compounds **D2, D10, D11, F9, F10, F11, G3-G7, G10, H2, H5, J5**, and **J6** were the least selective but the others revealed reasonable selectivity toward the tumorigenic cell.

### Pharmacophore modeling

Elucidation of the binding approaches for the synthesized compounds is suggested based on finding the active structures. Table 1 shows the structure of the training set compounds **(A1, A9, B1, B5, B9, C3, D5, E3, E5, F3, F5, G3, G5, H5, I3**, and **J5)** as well as the test set compounds **(B7, B8, B10, D2-D4, E2, F2, H8, J4**, and **J8)**. Based on the assumption that the active compounds bind in a similar fashion at the active site. Ligandscout program [33] was employed to evaluate the common features essential for antiproliferative activity and the hypothetical geometries adopted by these ligands in their most active forms. Thus, these compounds were submitted for pharmacophore model generation based on the shared chemical features. Diverse conformations within 20 kcal/mol energy range were generated and submitted to the alignment procedure.

**Figure 1:**
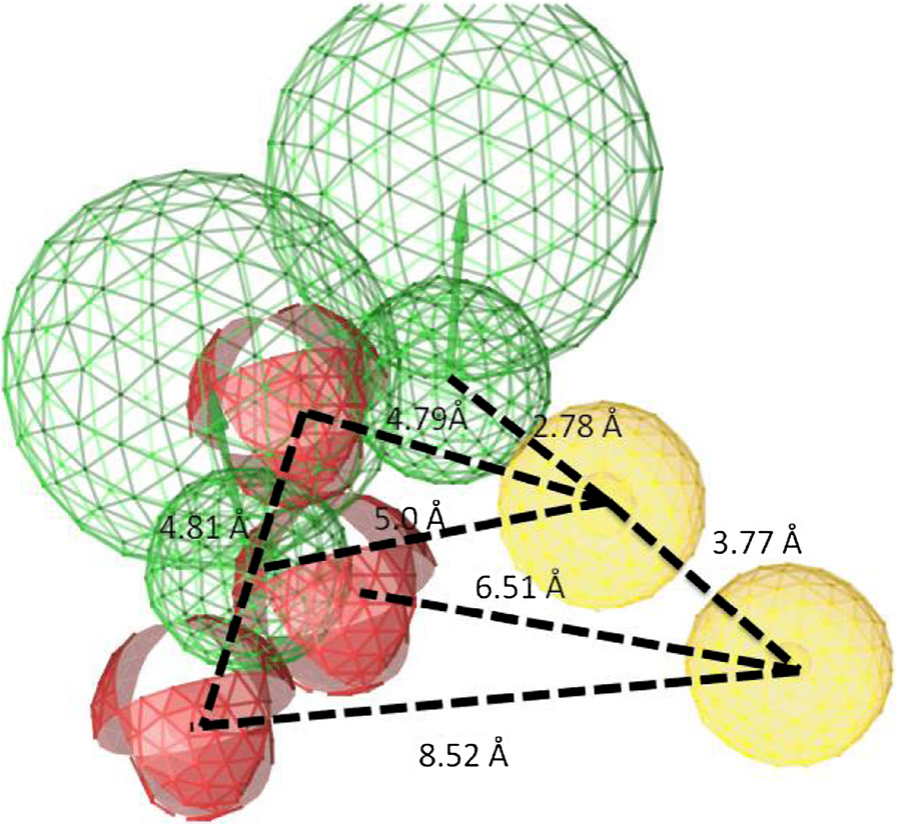
Proposed pharmacophore model for antiproliferative activity (red, HBA; yellow, hydrophobic; green, HBD).

The successful pharmacophore run resulted in generation of 10 hypotheses (Hypo1-10, Table 3). Hypo1-7 composed of two hydrophobes, three hydrogen bond acceptors and two hydrogen bond donors. According to its highest rank score and mapping into all training set molecules, hypo1 was considered statistically as the best hypothesis and was selected for further investigation and analysis. The top-ranked chemical feature-based pharmacophore model identified in this study is shown in Figure 1. This pharmacophore model contains seven chemical features: two hydrophobes (orange), three hydrogen bond acceptors (red) and two hydrogen bond donors (green).

**Table 3:** Summary of the generated pharmacophores of the Antiproliferative activities of the synthesized Schiff bases against MCF-12A cell line

**Hypothesis**	Features^*^	**Rank score**
Hypo1	HHAAADD	0.8665
Hypo2	HHAAADD	0.8657
Hypo3	HHAAADD	0.8656
Hypo4	HHAAADD	0.8652
Hypo5	HHAAADD	0.8651
Hypo6	HHAAADD	0.8624
Hypo7	HHAAADD	0.8622
Hypo8	HHRAAADD	0.8578
Hypo9	HHRAAADD	0.8550
Hypo10	HHRAAADD	0.8483

^*^H hydrophobic, *R* aromatic ring, *A* hydrogen bond acceptor, *D* hydrogen bond donor.

**Figure 2:**
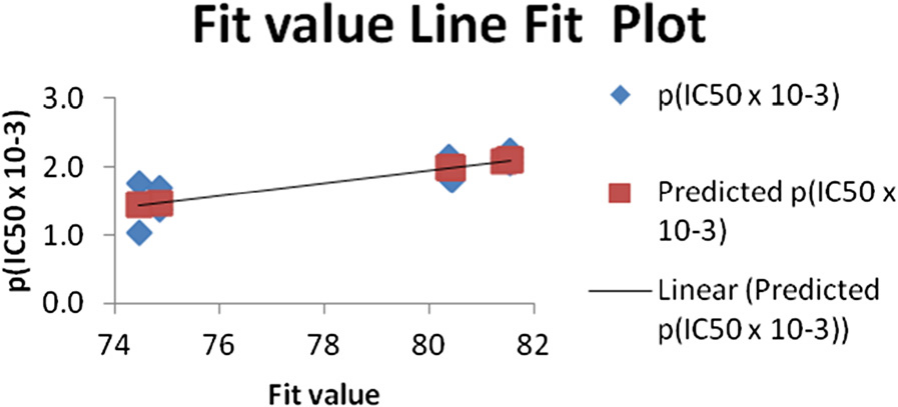
**Fit plot of the predicted pIC**_**50 **_**of the training set compounds with its experimental pIC**_**50.**_

**Figure 3:**
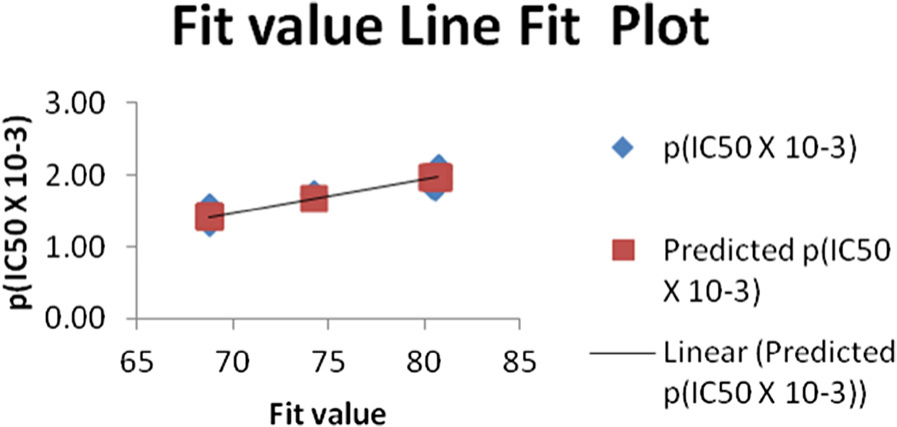
**Fit plot of the predicted pIC**_**50 **_**of the test set compounds with its experimental pIC**_**50.**_

All the training set and test set compounds were mapped onto hypo1 with scoring the orientation of a mapped compound within the hypothesis features using a “fit value” score. As a quick and primary validation of hypo1, mapping of the compounds found to show a good agreement between the fit value and the biological activity (Tables 4 and 5, Figures 2 and 3).

**Table 4:** Output for Hypo1 mapping and predictive model of training set compounds

**Compds**	**IC**_**50**_** (μM)**	**p(IC**_**50**_** × 10**^**-3**^)	**Fit value**	**Predicted p(IC**_**50**_** × 10**^**-3**^)	**Residuals**
**A1**	20.04	1.70	74.84	1.47	0.23
**A9**	90.67	1.04	74.46	1.44	-0.40
**B1**	42.30	1.37	74.84	1.47	-0.10
**B5**	7.30	2.14	81.42	2.09	0.05
**B9**	17.42	1.76	74.46	1.44	0.32
**C3**	14.86	1.83	80.39	1.99	-0.16
**D5**	8.67	2.06	81.54	2.10	-0.04
**E3**	7.13	2.15	80.37	1.99	0.16
**E5**	10.30	1.99	80.42	2.00	-0.01
**F3**	14.00	1.85	80.37	1.99	-0.14
**F5**	10.40	1.98	80.42	2.00	-0.02
**G3**	9.88	2.01	80.44	2.00	0.01
**G5**	6.50	2.19	81.52	2.10	0.09
**H5**	5.88	2.23	81.54	2.10	0.13
**I3**	15.81	1.80	80.44	2.00	-0.20
**J5**	6.99	2.16	81.54	2.10	0.06

**Table 5:** Output for Hypo1 mapping and predictive model of test set compounds

**Compds**	**IC**_**50**_** (μM)**	**p(IC**_**50**_** × 10**^**-3**^)	**Fit value**	***Predicted* p(IC**_**50**_** × 10**^-3^)	***Residuals***
**B7**	47.25	1.33	68.76	1.30	0.03
**B8**	27.23	1.56	68.78	1.30	0.26
**B10**	17.88	1.75	74.2	1.58	0.17
**D2**	8.30	2.08	80.71	1.92	0.16
**D3**	13.86	1.86	80.39	1.90	-0.04
**D4**	9.98	2.00	80.56	1.91	0.09
**E2**	7.76	2.11	80.71	1.92	0.19
**F2**	12.90	1.89	80.71	1.92	-0.03
**H8**	40.65	1.39	68.78	1.30	0.09
**J4**	14.52	1.84	80.56	1.91	-0.07
**J8**	45.51	1.34	68.78	1.30	0.04

Initial investigation of the results shown in Tables 4 and 5 revealed a moderate correlation between the fit value and the biological activity of each of the tested compounds. The highly active compounds showed a range of fit value of 81.54-80.03 where the moderately active derivatives showed a lower fit value average of 74.0. This initial correlation encouraged us to generate a linear model based on “fit value” to predict the biological activity of the compounds under investigation. The generated model (Equation 1) showed good statistics and was used successfully to calculate the activity of the tested compounds (Table 4).

(1) $$\;{\text{pIC}}_{50}\text{=}7.4164\;\text{fit}\;\text{value+}65.291$$

n = 16, st. error = 0.181, R = 0.834, R² = 0.696 Where n: number of compounds; R: multiple correlation coefficient

**Figure 4:**
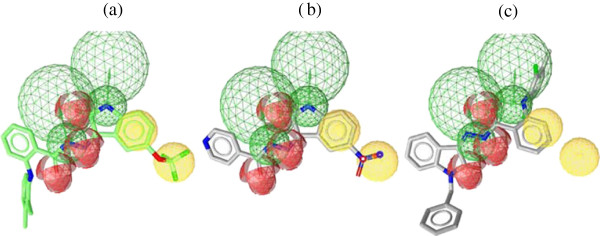
(**a**) **Best aligned pose of compound H5 (IC**_**50**_ **= 5.88 μM) superposed with the query (Hypo1).** (**b**) Best aligned pose of compound **A6** (IC_50_ > 100 μM) fitted inadequately with the query (Hypo1). (**c**) Best aligned pose of weak **I8** (IC_50_ > 100 μM) overlaid onto the pharmacophore model (Hypo1).

Figures 4a-c showed the alignment of the hypothesis model with compounds **H5, A6,** and** I8** as representative examples. A closer look at the mapped structures revealed the importance of certain structural features for activity. The substituted benzene ring of the isatin scaffold is thought to be critical for activity where the slight displacement of its fused benzene ring away from hydrophobic pharmacophore center (Figure 4b) or displacement of the isatin nitrogen away from the hydrogen bond donor pharmacophore center (Figure 4c) can partially explain their lack of activity. Furthermore, lack of antiproliferative activity of the derivatives containing 5-unsubstituted isatin moiety **A1-K1** could be attributed to their missing of one of the essential hydrophobic pharmacophore centers (Figure 4c). The rest of the features that are common for all compounds are the oxygen atom at 2-position of the isatin, the hydrazone nitrogen as hydrogen bond acceptor, and the hydrazide NH as hydrogen bond donor.

### Docking procedure

**Figure 5:**
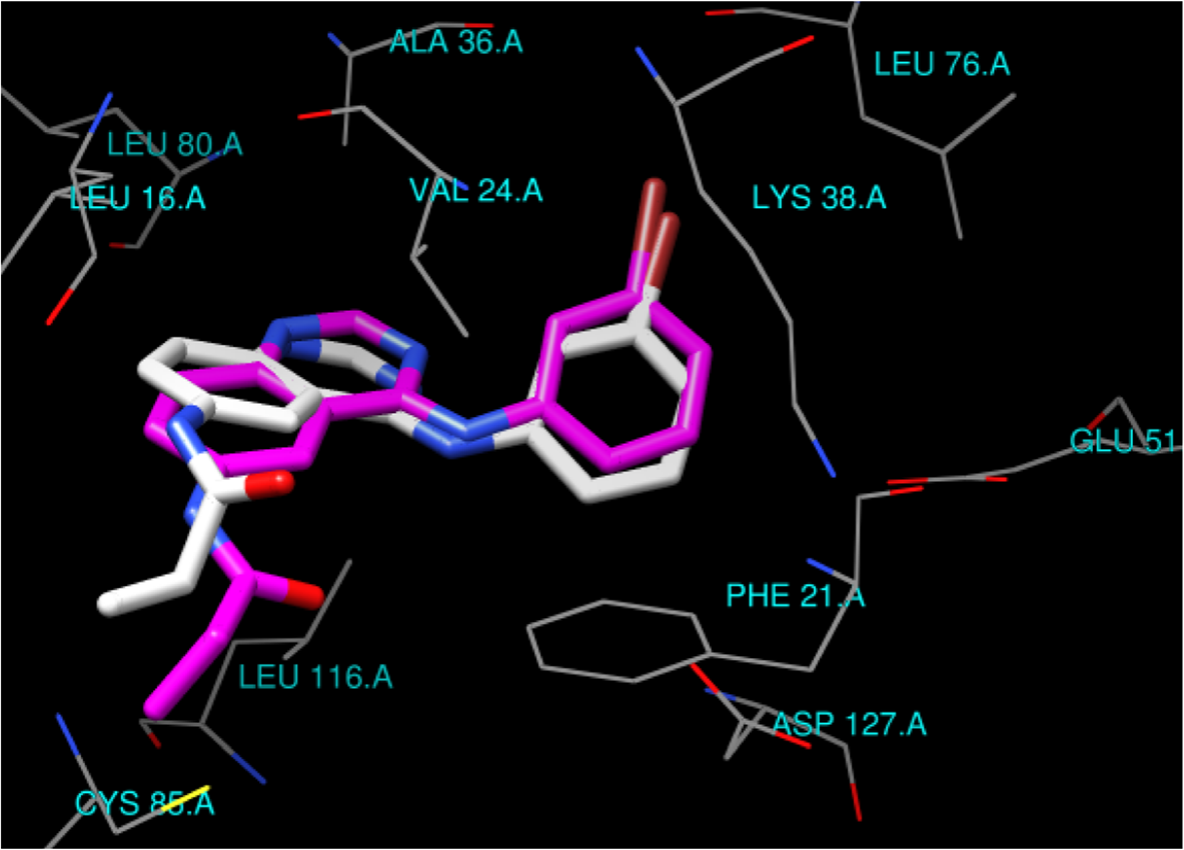
***N*-[4-(3-bromophenylamino)quinazolin-6-yl]acrylamide (DJK_3021_A) from 2J5F (X-ray magenta, docked white) oriented epidermal growth factor receptor (EGFR) kinase binding site.**

Docking study was undertaken using Dock6.4 [34] in order to investigate the possible interactions between the designed compounds and the active site of the epidermal growth factor receptor (EGFR) kinase and to compare it with the binding mode of the known EGFR inhibitor *N*-[4-(3-bromophenylamino)quinazolin-6-yl]acrylamide (DJK_3021_A). The X-ray structure of the enzyme bounded with DJK_3021_A was taken from the protein data bank; PDB code: 2J5F [35]. The RMSD value difference of 1.005 Å of the pose of the non-restricted redocking of the X-ray structure of the EGFR inhibitor (DJK_3021_A) from itself also confirmed the approach (Figure 5). The binding site includes hydrophobic pocket delineated by the side chains of Leu16, Phe21, Val24, Ala36, Lys38, Glu51, Leu76, Leu80, Cys85, Leu116, Asp127 (Figure 5).

**Figure 6:**
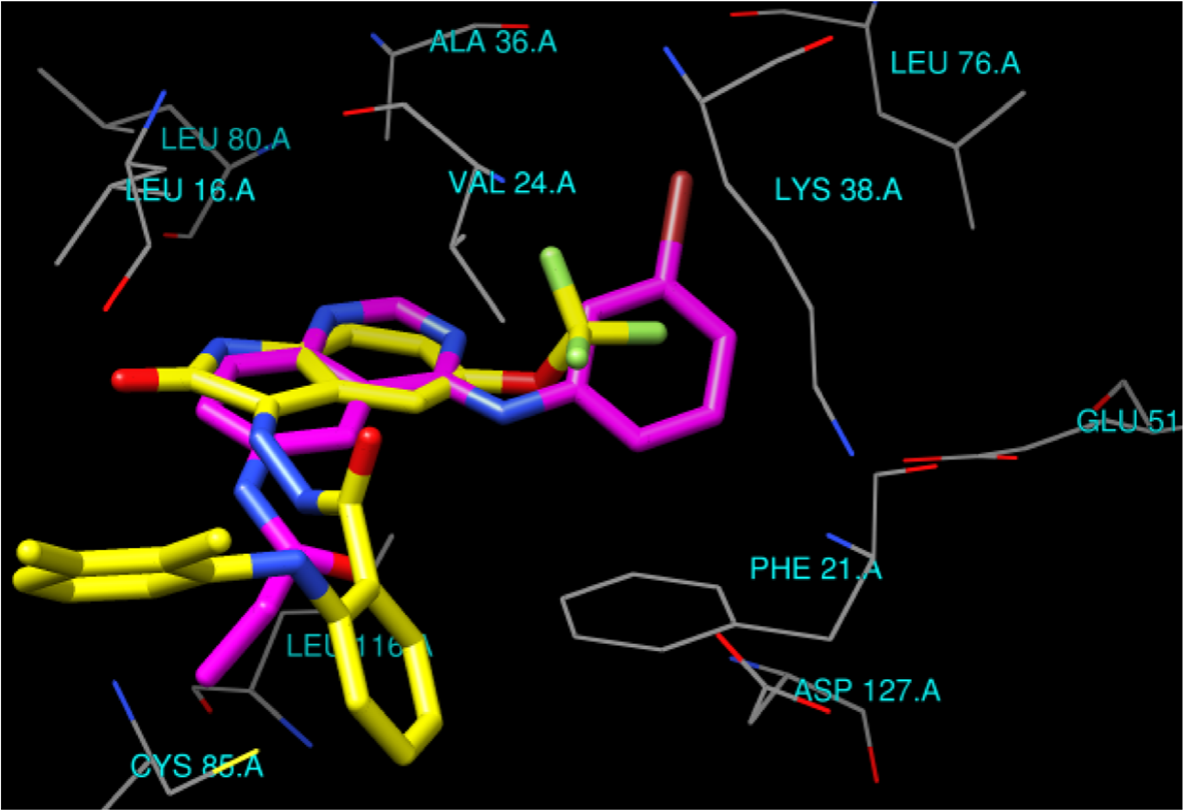
Compound H5 (colored Yellow) docked in EGFR binding site and overlaid with DJK_3021_A x-ray structure (colored magenta).

**Figure 7:**
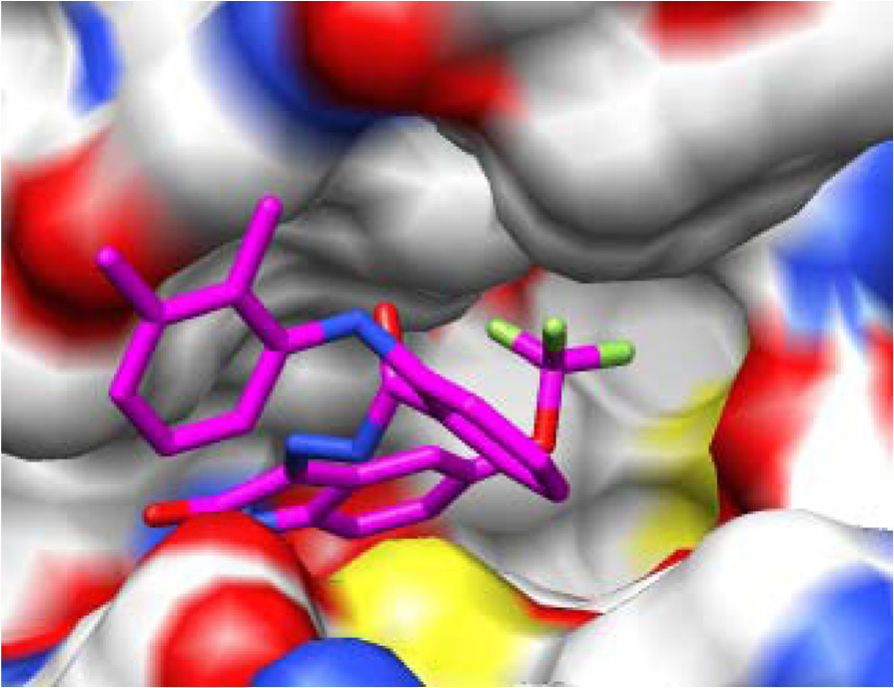
Binding site surface with docked, compound H5 (colored magenta).

The docking poses of compound **H5**, as an example of the designed compounds (Figures 6 and 7), showed that isatin scaffold structure is oriented in the binding site as the same as the quinazoline moiety of the DJK_3021_A X-ray structure with displacement of the hydrogen bond acceptor atom in compound **H5** from those of the quinazoline ring of the DJK_3021_A. The isatin scaffold is oriented in the hydrophobic pocket surrounded by the side chains of Leu16, Phe21, Leu80 and Leu116. The hydrophobic portion of trifluoromethoxy group is overlaid with the bromophenyl moiety of the DJK_3021_A whereas it is stabilized between Val24 and Lys38 with the hydrophobic interactions. Moreover, hydrophobic aromatic substituent at nitrogen atom of isatin in **H5** is aligned with the hydrophobic portion of acrylamide substituent of the DJK_3021_A whereas it is positioned in parallel orientation between its pi system and Leu116 with additional hydrophobic interaction between the aromatic ring, of *N*-benzoyl moiety, and Phe21 side chain.

Comparing the docking poses of the designed derivatives with DJK_3021_A, it could be postulated that the designed compounds might act on the same enzyme target where DJK_3021_A acted.

## Conclusions

A combinatorial library of 121 Schiff bases of indoline-2,3-dione (isatin) was investigated for their potential antiproliferative activity. Potent activity was observed with some of these derivatives against K562 chronic myelogenous leukemia cells with considerable selectivity compared with the non-tumorigenic MCF-12A cell line. Pharmacophore modeling study revealed that these compounds are able to effectively satisfy the proposed common feature sites using energy accessible conformers (E_conf_ < 20 kcal/mol). Also, docking study could suggest the similarity in binding mode of the designed compounds and DJK_3021_A with the EGFR kinase in its X-ray structure.

### Experimental

Isatins and hydrazides building blocks were obtained either commercially or synthesized along with the designed target Schiff bases according to the reported literatures [26,27,29-31]. Cytotoxicity was done at Stem Cell Therapy Program, King Faisal Specialized Hospital and Research Center, Riyadh-Saudi Arabia.

#### Cell cytotoxicity assay

K562 chronic myelogenous leukemia cells were purchased from the American Type Culture Collection. Cells were maintained in RPMI 1640 (Sigma), supplemented with 10% FCS (Cambrex Bio Science), 100 IU/mL penicillin, 100 mg/mL streptomycin and 2 mmol/L L-glutamine (Sigma) and were used to investigate the cytotoxicity of all the synthesized compounds.

MCF-12A cell line is a non-tumorigenic epithelial cell line established from tissue taken at reduction mammoplasty from a nulliparous patient with fibrocystic breast disease that contained focal areas of intraductal hyperplasia and were used to investigate the cytotoxicity of all the synthesized compounds.

Cells were seeded into 96-well plates at 0.4*10^4^/well and incubated overnight. The medium was replaced with fresh one containing the desired concentrations of the compounds. After 48 h, 10 μl of the WST-1 reagent were added to each well and the plates were reincubated for 4 h at 37°C. The amount of formazan was quantified using ELISA reader at 450 nm.

### Selectivity index (SI)

In the present study, the degree of selectivity of the synthetic compounds is expressed as per the previous reports [36,37]: SI = IC_50_ of pure compound in a normal cell line/IC_50_ of the same pure compound in cancer cell line, where IC_50_ is the concentration required to kill 50% of the cell population.

### Molecular modeling

#### Ligand based pharmacophore modeling

The study was carried out using the software LigandScout (version 3.0). LigandScout program was used to derive the 3D chemical feature-based pharmacophores from the structural data of the synthesized compounds (Table 1) using default settings [33]. Compounds **A1, A6-A9, B1, B5-B10, C3, C6-C8, D2-D5, E2, E3, E5, F2, F3, F5, G3, G5, G9, H5, H8, I3, I8, J4, J5, J8**, and **K6** are included in the modeling method. Prior to the generation of pharmacophore hypotheses, the training set compounds (**A1, A9, B1, B5, B9, C3, D5, E3, E5, F3, F5, G3, G5, H5, I3**, and **J5**) were converted to 3D structure and were used to generate diverse conformations. Diverse Conformation Generation protocol implemented in LigandScout program was used to generate conformations using the best conformation model generation method. Other parameters like maximum number of 500 conformers, and an energy threshold value of 20 kcal/mol above the global energy minimum were chosen during conformation generation. During pharmacophore hypothesis generation four pharmacophoric features like hydrogen bond acceptor (HBA), hydrogen bond donor (HBD), ring aromatic (RA) and hydrophobic (HY) were selected based on the feature mapping results. All parameters were set to their default values.

#### Pharmacophore validation

The generated pharmacophore hypothesis was validated using leave-one-out and test set methods.

#### Leave-one-out method

The pharmacophore hypothesis is cross validated by leave-one-out method. In this method, one compound is left in the generation of a new pharmacophore model and its affinity is predicted using that new model. The model building and estimation cycle were repeated until each compound was left out once [38]. This test was performed to verify whether the correlation coefficient of the training set compounds is strongly depend on one particular compound or not [39].

#### Test set method

Compounds **B7, B8, B10, D2-D4, E2, F2, H8, J4**, and **J8** were selected as test set compounds. This method is used to elucidate whether the generated pharmacophore hypothesis is proficient to predict the activities of the compounds other than training set and classify them correctly in their activity scale. The conformation generation for test set compounds was carried out in a similar way like training set compounds using conformation analysis algorithm. The compounds associated with their conformations were subsequently carried out for pharmacophore mapping using Ligand Pharmacophore Mapping protocol with Best/Flexible Search option.

### Docking procedure

All molecular modeling studies were performed on PC windows Vista Home Premium Intel(R) Core(TM)2 Duo, 1.83 GHz using Dock 6.4 [34]. All compounds were generated in the protonation state under physiological condition. The coordinates of the X-ray structure of the epidermal growth factor receptor (EGFR) kinase domain in complex with an irreversible inhibitor DJK_3021_A (PDB code: 2J5F) was taken from the Protein Data Bank [35]. The co-crystallized ligand was docked in its original protein structure. Docking was performed with default settings to obtain a population of possible conformations and orientations for the ligands at the binding site. A 10 Å sphere around the centre of the binding pocket was defined as binding pocket for the docking runs. All torsion angles in each compound were allowed to rotate freely.

## Competing interests

The authors declare that they have no competing interests.

## Authors’ contributions

TA has formulated the research idea, result’s interpretation and discussion and prepared the manuscript, AAR undertook the molecular modeling studies, result’s interpretation and shared in preparation of the manuscript, MIA participated in result’s interpretation and shared in preparation of the manuscript, AA carried out antiproliferative investigations, HAA participated in preparation of the manuscript. All authors have read and approved the final manuscript.

## Supplementary Material

Additional file 1 Antiproliferative evaluation results of the synthesized combinatorial mixtures **M1-M22** and schematic representation of an orthogonal deconvolution for prediction of the active compounds. Click here for file
