# Supplementary material for: Schiff bases of indoline-2,3-dione (isatin) with potential antiproliferative activity
Source: Chem Cent J. 2012 May 30;6:49. doi: 10.1186/1752-153X-6-49 (PMC3523985; doi:10.1186/1752-153X-6-49)
Supplement: Additional file 1 — Antiproliferative evaluation results of the synthesized combinatorial mixtures M1-M22 and schematic representation of an orthogonal deconvolution for prediction of the active compounds. [file 1752-153X-6-49-S1.doc]

**Schiff bases of indoline-2,3-dione (isatin) with potential antiproliferative activity**

Tarek Aboul-Fadl, Awwad A. Radwan, Mohamed I. Attia, Abdullah Al-Dhfyan and Hatem A. Abdel-Aziz

| **Hydrazides X**  **Isatins Y** | | **A** | **B** | **C** | **D** | **E** | **F** | **G** | **H** | **I** | **J** | **K** |  |  |
| --- | --- | --- | --- | --- | --- | --- | --- | --- | --- | --- | --- | --- | --- | --- |
|  |  |  |  |  |  |  |  |  |  |  |  |  | **Set 1** | IC_50_^a^ (μM) |
| **1** |  | **A1** | **B1** | **C1** | **D1** | **E1** | **F1** | **G1** | **H1** | **I1** | **J1** | **K1** | **M1** | 7.1 ± 0.04 |
| **2** |  | **A2** | **B2** | **C2** | **D2** | **E2** | **F2** | **G2** | **H2** | **I2** | **J2** | **K2** | **M2** | 9.76 ± 0.027 |
| **3** |  | **A3** | **B3** | **C3** | **D3** | **E3** | **F3** | **G3** | **H3** | **I3** | **J3** | **K3** | **M3** | 7.16 ± 0.069 |
| **4** |  | **A4** | **B4** | **C4** | **D4** | **E4** | **F4** | **G4** | **H4** | **I4** | **J4** | **K4** | **M4** | 6.63 ± 0.02 |
| **5** |  | **A5** | **B5** | **C5** | **D5** | **E5** | **F5** | **G5** | **H5** | **I5** | **J5** | **K5** | **M5** | 4.53 ± 0.028 |
| **6** |  | **A6** | **B6** | **C6** | **D6** | **E6** | **F6** | **G6** | **H6** | **I6** | **J6** | **K6** | **M6** | 4.48 ± 0.015 |
| **7** |  | **A7** | **B7** | **C7** | **D7** | **E7** | **F7** | **G7** | **H7** | **I7** | **J7** | **K7** | **M7** | 5.09 ± 0.01 |
| **8** |  | **A8** | **B8** | **C8** | **D8** | **E8** | **F8** | **G8** | **H8** | **I8** | **J8** | **K8** | **M8** | b |
| **9** |  | **A9** | **B9** | **C9** | **D9** | **E9** | **F9** | **G9** | **H9** | **I9** | **J9** | **K9** | **M9** | 9.29 ± 0.019 |
| **10** |  | **A10** | **B10** | **C10** | **D10** | **E10** | **F10** | **G10** | **H10** | **I10** | **J10** | **K10** | **M10** | 9.76 ± 0.018 |
| **11** |  | **A11** | **B11** | **C11** | **D11** | **E11** | **F11** | **G11** | **H11** | **I11** | **J11** | **K11** | **M11** | 14.57 ± 0.05 |
| **Set 2** | | **M12** | **M13** | **M14** | **M15** | **M16** | **M17** | **M18** | **M19** | **M20** | **M21** | **M22** |  | |
| IC_50_^a^ (μM) | | 12.02 ±  0.026 | b | 22.6 ±  0.038 | 4.85 ±  0.018 | 10.1 ±  0.05 | b | 9.64 ±  0.003 | 21.06 ±  0.02 | b | b | 4.51 ±  0.003 |  |  |

Antiproliferative evaluation results of the synthesized combinatorial mixtures **M1-M22** and schematic representation of an orthogonal

deconvolution for prediction of the active compounds.

^a^ IC_50_: concentration of the compound (μM) producing 50% cell growth inhibition after 48 h of compound exposure, as determined by the WST-1 assay. Each experiment was run at least two times, and the results are presented as average values ± standard deviation. ^b^Compounds or mixtures having IC_50_ value > 100 μ
